# Cytokines from SARS-CoV-2 Spike-Activated Macrophages Hinder Proliferation and Cause Cell Dysfunction in Endothelial Cells

**DOI:** 10.3390/biom14080927

**Published:** 2024-07-30

**Authors:** Giulia Recchia Luciani, Amelia Barilli, Rossana Visigalli, Valeria Dall’Asta, Bianca Maria Rotoli

**Affiliations:** Laboratory of General Pathology, Department of Medicine and Surgery, University of Parma, 43126 Parma, Italy; giulia.recchialuciani@unipr.it (G.R.L.); amelia.barilli@unipr.it (A.B.); rossana.visigalli@unipr.it (R.V.); biancamaria.rotoli@unipr.it (B.M.R.)

**Keywords:** COVID-19, cytokines, endothelial dysfunction, macrophages

## Abstract

Endothelial dysfunction plays a central role in the severity of COVID-19, since the respiratory, thrombotic and myocardial complications of the disease are closely linked to vascular endothelial damage. To address this issue, we evaluate here the effect of conditioned media from spike S1-activated macrophages (CM_S1) on the proliferation of human umbilical endothelial cells (HUVECs), focusing on the specific role of interleukin-1-beta (IL-1β), interleukin-6 (IL-6), interferon-gamma (IFN-γ) and tumor necrosis factor-alpha (TNF-α). Results obtained demonstrate that the incubation with CM_S1 for 72 h hinders endothelial cell proliferation and induces signs of cytotoxicity. Comparable results are obtained upon exposure to IFN-γ + TNF-α, which are thus postulated to play a pivotal role in the effects observed. These events are associated with an increase in p21 protein and a decrease in Rb phosphorylation, as well as with the activation of IRF-1 and NF-kB transcription factors. Overall, these findings further sustain the pivotal role of a hypersecretion of inflammatory cytokines as a trigger for endothelial activation and injury in the immune-mediated effects of COVID-19.

## 1. Introduction

Growing evidence supports the pivotal role of endothelial dysfunction (ED) in the pathogenesis of COVID-19 and in the progression to severe late complications [1]. These include lung injury, myocardial dysfunction, venous thromboembolism and systemic vasculitis, which actually share a common basis of ED. Hallmarks of these events are alterations of the vascular equilibrium toward vasoconstriction, systemic inflammation with associated tissue edema and onset of a procoagulant state, not only in the pulmonary vasculature, but throughout the body. As a result, severe COVID-19 is currently considered a hyperinflammatory prothrombotic disease with multiorgan involvement affecting the entire vasculature [2,3]. Similarly, ED has recently emerged as one of the many factors responsible for Post-COVID-19 Syndrome [4]. 

Two main mechanisms are involved in the onset of endothelial dysfunction in COVID-19: a direct effect of SARS-CoV-2 infection in endothelial cells, and indirect damage secondary to a dysregulated immune response with excessive release of pro-inflammatory mediators. As in other systemic inflammatory pathologies, indeed, a hyperproduction of inflammatory cytokines, namely “cytokine storm”, is known to play a pivotal role in COVID-19-associated endothelial injury [1]. These events, in the lung, can lead to a severe impairment of the pulmonary endothelium, resulting in hyperpermeability, which is the leading cause of pulmonary edema in COVID-19-associated acute respiratory distress syndrome, ARDS [5,6,7]. ARDS is, indeed, a heterogenous syndrome characterized by damage of the lung microvascular and alveolar epithelial cell barriers, vascular leakage, alveolar edema, hypoxia, and pulmonary fibrosis [8].

Among the pro-inflammatory cytokines observed in COVID-19 patients, interleukin-6 (IL-6), interleukin-1-beta (IL-1β), interferon-gamma (IFN-γ) and tumor necrosis factor-alpha (TNF-α) are generally associated with illness progression [9]. These cytokines, among their numerous functions, deserve particular attention since they are known to trigger endothelial activation and dysfunction [10,11,12]. Typical features of activated endothelial cells are the exposure of adhesion molecules that in COVID-19 mediate endotheliitis, as well as the induction of pro-coagulation factors, such as von Willebrand factor and plasminogen activator inhibitor-1 (PAI-1), that play important roles in the abnormal coagulation associated with COVID-19. Actually, a strong association between abnormal circulating vascular markers in plasma and poor prognosis of COVID-19 has been suggested [13].

In a previous contribution, we recently addressed the effect of the spike S1 protein from SARS-CoV-2 on the activation of human lung microvascular endothelial cells (HLMVECs) [14]. There, we demonstrated that inflammatory mediators released by spike-treated macrophages strengthen endothelial cell activation, by stimulating the expression of adhesion molecules, pro-coagulant markers, and chemokines, likely contributing to the impairment of vascular integrity. To further address this issue, we here focus on the effects of conditioned medium from S1-treated macrophages on endothelial cell viability and proliferation, and on the inflammatory mediators specifically involved in these effects.

## 2. Materials and Methods

### 2.1. Cell Culture and Experimental Treatments

Human monocytes were obtained as previously described [15] from buffy coats of healthy donors, enrolled by the Unit of Immunohematology and Transfusion of the Azienda Ospedaliero-Universitaria of Parma. The protocol of the study received the approval of the local ethical committee (460/2021/TESS/UNIPR) and was conducted in accordance with the principles of the Declaration of Helsinki (1964). Adherent monocytes were maintained for 5 days in RPMI 1640 medium supplemented with 10% endotoxin-free fetal bovine serum (FBS) and 50 ng/mL of recombinant human Granulocyte Mϕ-Colony-Stimulating Factor (GM-CSF, RELIATech by Vinci-Biochem, Vinci (FI), Italy) to obtain monocyte-derived macrophages (MDMs). MDMs were incubated in the absence and in the presence of 5 nM S1 subunit of SARS-CoV-2 spike recombinant protein (ARG70218; Arigo Biolaboratories, Taiwan), premixed with 2 µg/mL Polymyxin B to exclude any possible contamination by lipopolysaccharide (LPS). After 24 h, the incubation media of macrophages were collected as conditioned media from control MDMs (CM_cont) and S1-treated MDMs (CM_S1). For the experiments, CM and CM_S1 were obtained by pooling conditioned media from MDMs of 4 different donors. 

Human Umbilical Vein Endothelial Cells (HUVECs) were purchased from Thermo Fisher Scientific (Monza, Italy). Cells were routinely grown in Human Large Vessel Endothelial Basal Medium supplemented with Large Vessel Endothelial Supplement (LVES), according to the manufacturer’s instructions. Cells were employed between passages 1 and 6. For the experiments, HUVECs, seeded at the density of 5 × 10^4^ cells/mL in multiwell plates, were treated as required by the experimental plan 24 h after seeding. When incubating HUVECs with CM_cont or CM_S1, Large Vessel Endothelial Supplement (LVES) was added to provide HUVECs with supplements required for endothelial cell growth, lacking in RPMI 1640 medium. The effects of cytokines were, instead, assessed by incubating HUVECs in complete culture medium supplemented with 5 ng/mL of IFN-γ or IL-1β, or 50 ng/mL of TNF-α or IL-6 (R&D by Bio-Techne, Milano, Italy), used either alone or combined. 

### 2.2. Cell Proliferation 

HUVECs, seeded in 48-well plates, were treated as required. After trypsinization, cell proliferation was determined by counting the number of adherent cells with a Cell Counter ZM (Coulter Electronics Ltd., Luton, UK). 

### 2.3. Cell Cycle Analysis

For the analysis of cell cycle, HUVECs, seeded in 12-well plates, were treated for 48 h as required by the experimental plan. After trypsinization, cells were collected and incubated in a hypotonic solution containing 0.1% sodium citrate, 0.5% NP40, 10 μg/mL RNase A and 20 μg/mL propidium iodide (PI). After 18 h at 4 °C, cell cycle distribution was assessed with a Cytoflex flow cytometer (Beckman Coulter, Milano, Italy). 

### 2.4. Cell Death 

Apoptosis was measured by employing Annexin V FITC/Dead Cell Apoptosis Kit for flow cytometry (Thermo Fisher Scientific). After the treatment, both floating and adherent cells were washed with PBS and resuspended in 200 μL of binding buffer supplemented with Annexin-V FITC and propidium iodide (PI), according to the manufacturer’s instructions. After incubation for 20 min at RT in the dark, 300 μL of binding buffer was added, and samples were analyzed with a Cytoflex flow cytometer (Beckman Coulter). In scatter plots, cells that were negative for both PI and Annexin V are considered healthy (Q1-LL), while early and late apoptotic cells were identified as PI negative/Annexin V positive (Q1-LR), and Annexin V and PI positive (Q1-UR), respectively. PI positive/Annexin V negative cells were considered necrotic cells (Q1-UL). 

### 2.5. Western Blot Analysis

Protein expression was analyzed in cell lysates obtained as already described [16]. After separation on Bolt™ 4–12% Bis-Tris mini protein gel (Thermo Fisher Scientific), proteins were transferred to PVDF membranes (Immobilon-P membrane, Thermo Fisher Scientific). After incubation for 60 min in blocking solution (4% non-fat dried milk in TBST, Tris-buffered saline solution +0.5% Tween), proteins were labelled through overnight incubation at 4 °C with anti-phospho-NF-κB p65 (Ser536), anti-phospho-IκBα (Ser32/36), anti-phospho-STAT1 (Tyr701), anti-IRF1, and anti-p21^WAF^ rabbit polyclonal antibody (1:2000, Cell Signaling Technology, Euroclone, Pero (MI), Italy) or with anti-vinculin mouse monoclonal antibody (1:2000, Merck), employed as loading control. Protein detection was then performed through incubation with secondary antibodies conjugated with horseradish peroxidase (HRP)-conjugated (anti-rabbit and anti-mouse IgG, Cell Signaling Technology; 1:10,000), detected with SuperSignal™ West Pico PLUS Chemiluminescent HRP Substrate (Thermo Fisher Scientific). Western blot images, taken with an iBright FL1500 Imaging System (Thermo Fisher Scientific), were analyzed with iBright Analysis Software (version 5.3).

### 2.6. RT-qPCR Analysis

Total RNA was isolated from cells seeded in 24-well plates with the GeneJET RNA Purification Kit (Thermo Fisher Scientific). After reverse transcription with the RevertAid First Strand cDNA Synthesis Kit (Thermo Fisher Scientific), gene expression was analyzed with a StepOnePlus Real-Time PCR System (Thermo Fisher Scientific) by employing specific primers (Table 1) and SYBR™ Green Master Mix (Thermo Fisher Scientific). The levels of the genes of interest was calculated with the ∆∆Ct method and expressed, relative to RPL15, as fold change of control cells (=1).

### 2.7. Statistical Analysis

Statistical significance was calculated with the Kruskal–Wallis test with Dunn’s test for multiple comparison or with the Mann–Whitney test, as specified in each Figure’s legend, by using GraphPad Prism 9 (GraphPad Software, San Diego, CA, USA). *p*-values < 0.05 were considered statistically significant.

### 2.8. Materials

Recombinant human cytokines were purchased from R&D: HEK293 expressed IFN-γ; E. coli-derived IL-1β/IL-1F2 protein; HEK293 expressed TNF-α; HEC293 expressed IL-6. Endotoxin-free fetal bovine serum was from Thermo Fischer Scientific, while Merck (Milano, Italy) was the source of all other chemicals.

## 3. Results

**Figure 1:**
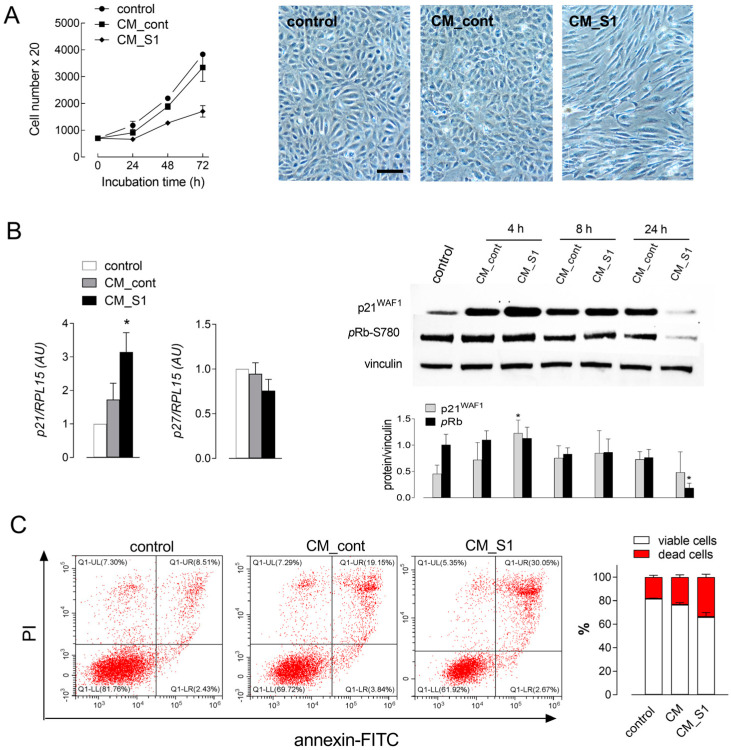
HUVECs were maintained in growth medium (control) or treated with conditioned medium obtained by incubating monocyte-derived macrophages in the absence (CM_cont) or in the presence of 5 nM S1 (CM_S1). (**A**) Cell proliferation was determined as specified in Section 2 at the indicated incubation times. Each point represents the media ± SD of three determinations in a representative experiment that, repeated three times, gave comparable results. Right: phase contrast microscopy images of cells treated for 72 h are shown. Bar = 100 µM. (**B**) After 4 h, the expression of cell cycle inhibitor p21 and p27 mRNA (left) was measured by means of RT-qPCR and expressed as fold change of control cells (=1). Bars are means ± SEM of four independent experiments, each performed in duplicate. * *p* < 0.05 vs. control with Kruskal–Wallis test. At the indicated times, the expression of p21^WAF1^ and phosphorylated Rb proteins was evaluated with Western blot analysis (right), as described in Section 2. Representative blots are shown, along with the mean ± SD of the densitometry analysis of three different experiments. * *p* < 0.05 vs. control cells with Kruskal–Wallis test. (**C**) After 48 h treatment, cell death was assessed with flow cytometry upon staining with Annexin V-FITC/Propidium iodide (see Section 2). Representative scatter plots are shown with the indicated % of viable and apoptotic/necrotic cells of 10,000 events. The experiments were replicated three times with comparable results; right panel shows the mean ± SD of viable (LL) and dead (UL + UR + LR) cells in the three experiments.

To investigate the immune-mediated effects of SARS-CoV-2 spike S1 protein on endothelial integrity, first, we exposed human endothelial cells (HUVECs) to conditioned medium (CM) obtained from untreated macrophages (CM_cont) or from S1-treated macrophages (CM_S1) and determined cell proliferation by counting cells at different time-points. The results obtained demonstrate that while the treatment with CM_cont caused only a modest reduction in cell growth compared to cells maintained under normal culture conditions, the incubation with CM_S1 severely hindered cell proliferation, halving the number of cells compared to CM_cont after 72 h of incubation (Figure 1A, left). 

At this time, CM_S1 also caused relevant changes in cell morphology, clearly evidenced by images obtained with phase contrast microscopy (Figure 1A, right). Cells incubated with CM_S1, indeed, appeared sparser compared to control conditions, and lost the polygonal morphology typical of endothelial cells to acquire a more spindle-like shape. No change was observed in cells treated with CM_cont, in terms either of confluence or morphology. 

To better define the mechanisms involved in the observed reduction in cell population, we next measured the expression of cell-cycle related genes and proteins. For this purpose, we first addressed the mRNA level of two well-known inhibitors of cell cycle progression, namely p21 and p27 [17]. Upon incubation with CM_S1, a significant induction of p21, but not of p27, mRNA was detectable compared to CM_cont-treated cells (Figure 1B). Consistently, a transient increase in p21 protein was evident just after 4 h of incubation with CM_S1, although a slight increase was observed also with CM_cont. After 24 h in the presence of CM_S1, the expression of p21 returned to basal level, while Rb protein, central regulator of the cell cycle [18], appeared hypo-phosphorylated. Hence, the decrease in cell number observed under this condition was likely related to an arrest of cell cycle, due to hypo-phosphorylation of Rb mediated by the induction of p21 protein. 

In parallel, the hypothesis that CM_S1 may exert cytotoxic effects was also explored. To this end, the occurrence of cell death was investigated with flow cytometry using Annexin V/Propidium iodide staining (Figure 1C). Results obtained indicate that the exposure of HUVECs to CM_cont for 48 h was responsible for an evident reduction in viable cells compared to the control population (76.6 ± 1.78% vs. 80.8 ± 0.48%), along with a significant increase in late apoptotic cells (16.6 ± 2.06% vs. 11.8 ± 1.6%). The induction of cell death, however, was much more appreciable when HUVECs were incubated with CM_S1; under this condition, indeed, the amount of viable cells further decreased, to 66.2± 3.7%, and the percentage of late apoptotic cells rose to 27.9 ± 2.6%, indicating that CM_S1 induces clear signs of endothelial cell death in vitro. Consistently, the quote of dead cells increased from 23.4 ± 2% to 33.8 ± 2.6%.

**Figure 2:**
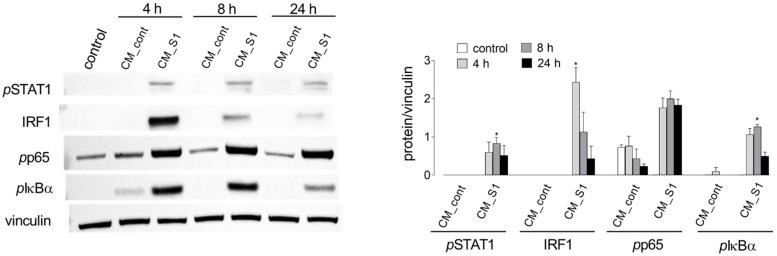
HUVECs were maintained in growth medium (control) or incubated in CM_cont or in CM_S1 for the indicated times. Protein expression was assessed by means of Western blot analysis, as detailed in Section 2. Representative blots are shown (**left**); mean ± SD of the densitometry analysis of three different experiments is also shown (**right**). * *p* < 0.05 vs. control cells with Kruskal–Wallis test.

In our previous contributions, we have evidenced the activation of NF-kB transcription factor by CM_S1 in A549 alveolar epithelial cells [19], as well as the primary role played by IFN-regulatory factor 1 transcription factor in the modulation of cell cycle [16]. In light of those findings, we here evaluated whether the impairment of endothelial viability observed under our experimental conditions was referable to the activation of STAT1/IRF-1 and NF-κB pathways. Results shown in Figure 2 clearly indicate that neither IRF-1 nor STAT1 were activated when endothelial cells were maintained in control medium or exposed to CM_cont; instead, the incubation of HUVECs with CM_S1 caused an evident, transient induction of IRF-1 protein and a significant increase in the phosphorylation of STAT1. Under the same condition, a stable activation of NF-κB also occurred, as demonstrated by the phosphorylation of the p65 subunit of the transcription factor, as well as of the inhibitor IκBα.

**Figure 3:**
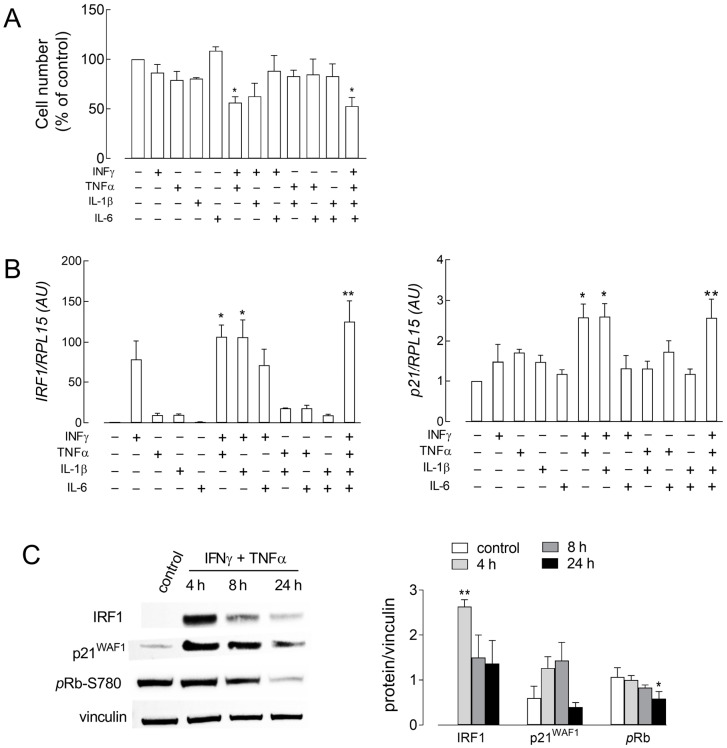
HUVECs were incubated in growth medium, either in the absence (control) or in the presence of the indicated cytokines. (**A**) After 72 h, the number of adherent cells was measured to calculate cell proliferation, as described in Section 2. Data are expressed as percentage of control, untreated cells; each bar represents the media ± SEM of three experiments, each performed in triplicate. * *p* < 0.05 vs. control, untreated cells with Kruskal–Wallis test. (**B**) The expression of IRF-1 and p21 mRNAs was measured after 4 h of incubation with RT-qPCR. Data are expressed as fold change of control (=1). Bars are means ± SEM of three independent experiments, each performed in duplicate. * *p* < 0.05, ** *p* < 0.01, vs. control, untreated cells with Kruskal–Wallis test. (**C**) HUVECs were maintained in the absence (control) or in the presence of IFN-γ + TNF-α. The amount of the indicated proteins was assessed with Western Blot analysis, as detailed in Section 2. Representative blots are shown (left), along with mean ± SD of the densitometry analysis of three different experiments (right). * *p* < 0.05, ** *p* < 0.01, vs. control cells with Kruskal–Wallis test. Original Western Blot images can be found in the Supplementary Materials.

As previously demonstrated, the conditioned medium from S1-treated human macrophages is rich in many inflammatory mediators [16,20]. Among them, we here specifically focused on prototypical inflammatory cytokines TNF-α, IL-1β, IL-6 and IFN-γ so as to verify whether these mediators are involved in the observed effects of CM_S1. Hence, we next tested the effects of 50 ng/mL TNF-α or IL-6, and of 5 ng/mL IL-1β or IFN-γ, used alone or combined, on cell proliferation. The rationale for the concentrations adopted consists in the different amounts of these mediators in CM_S1, higher for TNF-α and IL-6 with respect to IL-1β and IFN-γ (see Supplementary materials Figure S1). As shown in Figure 3A, all mediators except IL-6 caused a mild, not significant reduction in cell number after 72 h of treatment when employed alone. Conversely, a marked decrease in endothelial population was observed upon exposure to IFN-γ combined with either TNF-α or IL-1β, with a reduction comparable to that induced by CM_S1. Under the same conditions, IRF-1 and p21 mRNA levels were also addressed (Figure 3B). The results obtained clearly indicate that the incubation with TNF-α, IL-1β or IL-6 had no effect on the expression of either gene. Instead, a significant induction of IRF-1 mRNA was observed after incubation with IFN-γ alone and, even more, when combined with TNF-α or IL-1β; the simultaneous presence of the three stimuli in the incubation medium did not further enhance the expression of the transcription factor. When considering p21, only the combination of IFN-γ with either of the two cytokines TNF-α or IL-1β caused a significant increase in p21 mRNA, which otherwise remained comparable to control conditions. Since TNF-α and IL-1β are known to activate the same signaling pathways, mainly involving NF-κB [21], we hereafter decided to limit our investigations to the effects of INF-γ + TNF-α, to avoid overlapping results. As shown in Figure 3C, the combined treatment with the two cytokines caused a marked and transient increase in both IRF-1 and p21^WAF1^ proteins, consistent with mRNA results; a concomitant, progressive decrease in Rb phosphorylation was also detected. 

**Figure 4:**
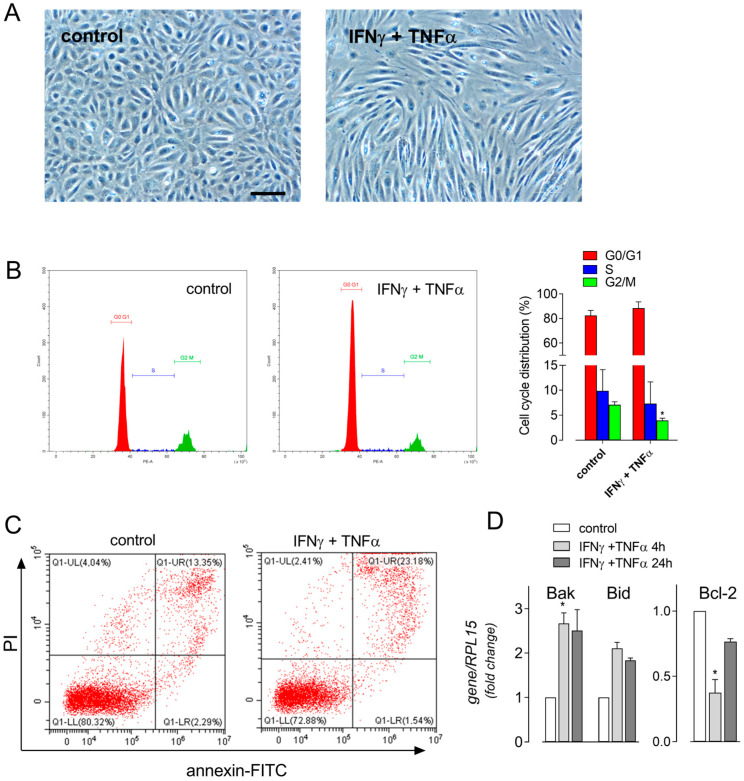
Cells were maintained in the absence (control) or in the presence of IFN-γ + TNF-α. (**A**) After 72 h, phase contrast microscopy images were taken (Bar = 100 µM). (**B**) After 24 h of incubation, cell cycle was analyzed with flow cytometry, as detailed in Section 2. Plots obtained in a representative experiment are shown (left), while cell distribution among the different phases of the cell cycle is shown (right), with bars corresponding to the mean ± SEM of data obtained in four independent experiments. * *p* < 0.05 vs. control cells with Mann–Whitney test. (**C**) After 48 h, apoptotic cell death was evaluated with flow cytometry through Annexin V-FITC/PI staining, as detailed in Section 2. Representative scatter plots are shown with the indicated % of viable and apoptotic/necrotic cells of 10,000 events. The experiments were replicated three times with comparable results. (**D**) At the indicated times, the expression of Bak, Bid and Bcl-2 mRNAs was measured by means of RT-qPCR. Data are expressed as fold change of untreated control cells (=1). Bars are means ± SEM of three independent experiments, each performed in duplicate. * *p* < 0.05 vs. control cells with Kruskal–Wallis test.

Also, the morphological changes induced by the incubation for 72 h with IFN-γ and TNF-α were similar to those observed in CM_S1-treated cells, with the appearance of elongated and sparse cells (Figure 4A). Under this condition, signs of both cell cycle arrest (Figure 4B) and cell death (Figure 4C) were evidenced with flow cytometry. The analysis of cell cycle showed, indeed, a small increase in the percentage of cells in G0/G1, compared to control cells (from 83 ± 2.4% to 88.5 ± 2.5%), paralleled by a significant decrease in cells in G2/M phases (from 7.1 ± 0.29% to 3.9 ± 0.23%); a modest but not significant decrease in cells in S phase was also observed (from 9.9 ± 2.1% to 7.3 ± 2.1%). As far as HUVEC viability is concerned, the incubation with IFN-γ + TNF-α significantly increased the percentage of late apoptotic population (from 11.8 ± 1.6% in untreated cells to 22.7 ± 2.1% upon cytokine exposure). The same treatment also modified the expression of known pro- and anti-apoptotic genes (Figure 4D); among the first, an induction of Bak and Bid was observed after both 4 and 24 h of incubation with the two cytokines, while a transient decrease in anti-apoptotic Bcl-2 was particularly evident after a treatment of 4 h.

## 4. Discussion

Endothelial dysfunction (ED) plays a central role in the severity of COVID-19, since the pathogenesis of the respiratory, thrombotic or myocardial complications of the disease is closely linked to vascular endothelial damage [22]. Moreover, increased risk of severe COVID-19 is more common in patients with comorbidities such as hypertension, diabetes, obesity, and cardiovascular disease, which are known to be associated with ED [23]. Several studies in this field support the hypothesis that endothelial injury in COVID-19 is ascribable to both a direct cytopathic effect of SARS-CoV-2 entry in endothelial cells and to an indirect uncontrolled inflammatory response, with high levels of circulating cytokines and other inflammatory mediators [24,25]. 

In this context, our previous study focused on the effects of supernatants from spike-activated macrophages (CM_S1) on endothelial cells and demonstrated that incubation for 24 h with this medium actually induces an inflammatory phenotype [14]. Now, by further addressing this issue on HUVECs, we show that the exposure to CM_S1 for 72 h significantly hinders cell growth and causes signs of endothelial cytotoxicity. Moreover, we also provide evidence that the cytokines present in this conditioned medium are responsible for the observed effects. We are aware that many mediators are present in the conditioned medium and may promote endothelial dysfunction; however, our findings clearly ascribe a role to IFN-γ and TNF-α in the attenuation of endothelial growth and in the induction of cell toxicity. To this concern, further loss-of-function studies using neutralizing antibodies to target these cytokines may definitely shed light on the issue.

These findings are consistent with several studies showing that inflammatory cytokines induce endothelial activation and dysfunction (for review, see [26]). Among them, accumulating in vivo and in vitro evidence confirm the pivotal role of TNF-α in endothelial activation and in vascular dysfunction, both in the macrovascular and microvascular circulation [27,28]. A critical role in the modulation of vascular inflammatory response has been also ascribed to IFN-γ; to this concern, the cytokine has been described to increase endothelial barrier permeability, likely through a remodeling of actin cytoskeleton and a reorganization of junctional proteins [29]. In addition, IFN-γ has been reported to induce cellular senescence in young HUVECs through a cell cycle arrest in G0/G1 and an up-regulation of p53 [30].

Really, in our hands, neither TNF-α nor IFN-γ exerted any relevant effect on endothelial cell proliferation when employed alone; conversely, the simultaneous exposure of HUVECs to the two mediators caused a decrease in cell viability comparable to that observed upon incubation with CM_S1. Under both experimental conditions, moreover, similar profound changes in cell morphology were observed, further sustaining a role for the two cytokines in the immune-mediated effects of spike protein.

A synergism between IFN-γ and TNF-α has been described in different models of endothelial cells. Lombardi et al. demonstrated, indeed, an increased secretion of many inflammatory mediators, both chemokines and cytokines, in human microvascular endothelial cells (HMEC-1) [31]. The same effects have been described also in human aortic endothelial cells (HAEC), where the combination of the two cytokines proved effective also in disrupting inter-endothelial junctions [32]. The same cocktail has been described to cause proliferative arrest and senescence in HUVECs [33], and, more recently, to aggravate endothelial damage caused in the same cells by CART123 effector cells [34]. Similarly, Karky et al. found that the simultaneous treatment with TNF-α and IFN-γ induces robust cell death in HUVECs [10], while Gomez et al. described the occurrence of pyroptotic cell death in ex vivo human corneal endothelial grafts [35]. In line with these studies, our results show that an apoptotic cell death actually occurs upon exposure to the combination of IFN-γ + TNF-α, as well as to CM_S1. 

By exploring the molecular mechanisms underlying the anti-proliferative/pro-apoptotic effects of CM-S1 and IFN-γ + TNF-α, we evidenced a significant modulation in the expression of p21 and Rb, suggesting a role for these proteins in the decrease in endothelial cell population. Both proteins are, indeed, well-characterized regulators of cell cycle progression [36], while p21 is known to have a role also in apoptotic cell death [37]. Similar results have been obtained by our group in A549 alveolar epithelial cells, where the same experimental conditions caused a marked arrest of cell growth mediated by an increase in p21 and a dephosphorylation of Rb protein through the activation of STAT/IRF-1 pathway [16,19]; in the same model, we recently demonstrated the involvement of both IRF-1 and NF-κB in the induction of iNOS by CM_S1 in A549 [38]. Here, we describe the activation of the same molecular pathways also in HUVECs. Although the JAK/STAT1/IRF-1 axis is a well-known target of IFN-γ, while NF-κB is among the transcription factors mostly involved in the cellular response to TNF-α, the two transcription factors are not really downstream targets of separate pathways. Indeed, a transcriptional synergism between NF-κB and STAT1 has been reported in the regulation of inflammatory gene expression [39,40]. Moreover, IRF-1 has been shown to mediate TNF-α signal transduction in endothelial cells [41,42], supporting the hypothesis of an interplay between the signaling pathways targeted by the two mediators. 

## 5. Conclusions

Overall, our findings ascribe to STAT/IRF-1 and NF-κB a role in the anti-proliferative/pro-apoptotic effects exerted, in endothelial cells, by spike-activated macrophages through the synergism of IFN-γ and TNF-α. These cytokines are hence postulated to cause endothelial dysfunction in COVID-19 and thus emerge as possible targets for preserving endothelial integrity in severe patients.

## Figures and Tables

**Table 1:** Sequences of the primer pairs employed for RT-qPCR analysis.

Gene/Protein Name (Gene ID)	Forward Primer	Reverse Primer
*BAK1*/BAK (578)	GAGATGGTCACCTTACCTCTGC	TCATAGCGTCGGTTGATGTCG
*BCL2*/Bcl-2 (596)	ATCGCCCTGTGGATGACTGAGT	GCCAGGAGAAATCAAACAGAGGC
*BID*/BID (637)	ATGGACCGTAGCATCCCTCC	GTAGGTGCGTAGGTTCTGGT
*CDKN1A*/p21 (1026)	CCTGTCACTGTCTTGTACCCT	GCGTTTGGAGTGGTAGAAATCT
*CDKN1B*/p27 (1027)	TAATTGGGGCTCCGGCTAACT	TGCAGGTCGCTTCCTTATTCC
*IRF1*/IRF1 (3659)	CTGTGCGAGTGTACCGGATG	ATCCCCACATGACTTCCTCTT
*RPL15*/RPL15 (6138)	GCAGCCATCAGGTAAGCCAAG	AGCGGACCCTCAGAAGAAAGC

## Data Availability

Data are available at https://osf.io/v5w3a/ accessed on 17 June 2024.

## Supplementary Materials

The following supporting information can be downloaded at: https://www.mdpi.com/article/10.3390/biom14080927/s1, Figure S1: Monocytes-derived macrophages (MDM) were incubated in the presence of 5 nM S1 pre-mixed with 2 μg/mL Polymyxin B. After 24 h, the indicated cytokines were quantified employing Quantikine™ ELISA kits (R&D Systems), according to the manufacturer’s instructions. Data are mean ± SD of results obtained in media from 4 different donors. Original Western Blot images can be found in the Supplementary Materials.
