# Supplementary material for: Cytokines from SARS-CoV-2 Spike-Activated Macrophages Hinder Proliferation and Cause Cell Dysfunction in Endothelial Cells
Source: Biomolecules. 2024 Jul 30;14(8):927. doi: 10.3390/biom14080927 (PMC11353037; doi:10.3390/biom14080927)
Supplement: Supplementary file 1 [file biomolecules-14-00927-s001.zip › biomolecules-3088207-supplementary.pdf]

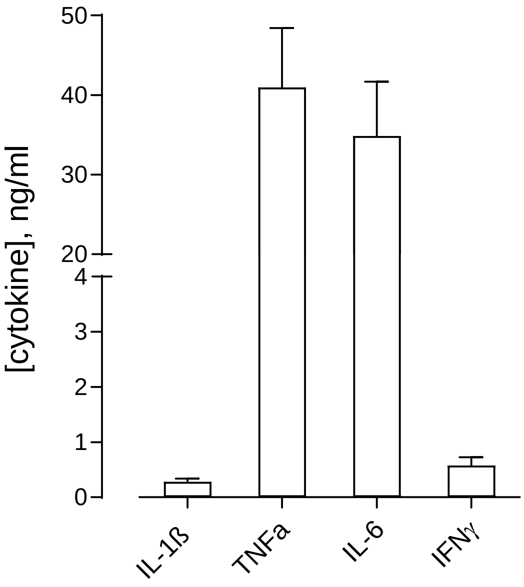

Figure 1S. Monocytes-derived macrophages (MDM) were incubated in the presence of 5 nM S1 pre-mixed with 2 μg/mL Polymyxin B. After 24 h, the indicated cytokines were quantified employing Quantikine™ ELISA kits (R&D Systems), according to the manufacturer's instructions. Data are mean ± SD of results obtained in media from 4 different donors.
